# Does Organic Disease of the Heart Preclude the Use of Chloroform in Parturition?1Read April 27, 1892, at the meeting of the Philadelphia County Medical Society.

**Published:** 1892-06

**Authors:** T. Ridgway Barker

**Affiliations:** Demonstrator of Obstetrics in the Medico-Chirurgical College, Philadelphia; Out-door Obstetrician to the Pennsylvania Dispensary


### DOES ORGANIC DISEASE OF THE HEART PRECLUDE THE USE OF CHLOROFORM IN PARTURITION?[1]

By T. RIDGWAY BARKER, M. D.,

Demonstrator of Obstetrics in the Medico-Chirurgical College, Philadelphia; Out-door Obstetrician to the Pennsylvania Dispensary.

In entering upon the discussion of a subject of such paramount importance to mother, offspring, and obstetrician, one cannot lay too much stress, at the very outset, upon the axiom that "A good remedy will fail of its effect if not properly administered." This fact must be kept uppermost in our mind if we would avoid fatal results, not due, however, to the employment of the agent, as some would make it appear, but to the lack of attention and care exercised in its administration. That there is a radical difference between surgical and obstetrical anesthesia (analgesia), goes without saying. If we consider for a moment the stages of anesthesia, which differ only in the profoundness of the impression—first, sopor; second, stupor; and, third, stertor—we cannot fail to notice that in analgesia one rarely has occasion to carry the effect beyond the first degree (sopor), while in the surgical variety we are obliged to advance beyond this and keep the patient in the second stage, or that of stupor, thus markedly increasing the gravity of the prognosis.

In this connection, let us devote a moment's consideration to the progressive effect of chloroform vapor upon the nerve-centers of the cerebro-spinal system, beginning, as it does, at the inferior extremity of the cord, sacro-lumbar, and gradually extending its paralyzing influence upward until it reaches and expends its force upon the medulla oblongata. These well-established clinical observations having been verified by physiological experiment, we are justified in putting them to practical use. What other agent, may be pertinently asked, can relieve—aye, abolish—pain so quickly and safely, yet leave reflex muscular contractility unimpaired, as chloroform? Ether and ethyl bromide have found favor with some practitioners, but neither can displace chloroform.

Fordyce Barker states in his writings: "I may say here that I have long regarded chloroform as the best and safest anesthetic in obstetrics, and that since 1850 I have used no other."

The danger from the employment of chloroform in this department of medicine depends more upon the carelessness with which

1. Read April 27, 1892, at the meeting of the Philadelphia County Medical Society.

it is administered than to any toxic effect inherent in it. The four cardinal points to be borne in mind when giving this anesthetic are: First, plenty of pure atmospheric air; second, liberation of a small amount of the vapor at a time; third, attention to the respiration; and, fourth, frequent observations as to the force and frequency of the cardiac action. That the recorded cases of death have been due, in a great measure, to saturation of the residual air in the lungs to a fatal degree can scarcely be doubted. A few deep, forced inspiratory efforts will quickly produce such a condition. Withdrawal of the agent, under these circumstances, cannot prevent the further entrance of the chloroform vapor into the circulation, for it already fills the air-cells. Nor will attempts at artificial respiration prove effectual, since but a small quantity of the residual air can be forced out of the lungs, while that which enters fails to sufficiently dilute the vapor, owing to the tardiness of diffusion. Let us not suppose, however, that because we administer to the parturient female small amounts of the drug continuously, therefore no risk is incurred, for experiments directed to solve this important question go to prove that even small doses, when continuously inhaled, tend to produce dangerous, and, at times, fatal cardiac exhaustion. Far different is the result when given intermittently, as is the unalterable rule in obstetrics. Should we seek authority for the statement that the dangers from the careful administration of chloroform in labor are too insignificant to warrant its refusal, we have only to turn to the *American System of Obstetrics* to find therein the following: "The danger, when chloroform is used only to the extent of mitigating or abolishing pain in childbirth, is practically *nil.*" Lusk, quoting from Bert's experiments, states "that chloroform might be intermittently administered for an indefinite period with safety." These remarks do not apply to its use in the third stage of labor, for, as is well known, after delivery of the child, it is likely to occasion relaxation of the uterus, thus favoring post-partum hemorrhage.

Offering the above as a preface to my remarks on the judiciousness of employing chloroform when the parturient female suffers from organic cardiac disease, it now remains for us to consider the effect of parturition upon this enfeebled circulatory organ, thereby securing a scientific basis for our conclusions. In the first stage of labor we find the muscular contractions confined to the uterine muscular layers, and directed toward overcoming the circular fibers of the cervix, while in the second or propulsive stage not only does the uterus exert its power to the utmost, but also the abdominal and respiratory muscles are brought into action by the will of the parturient in her efforts to expel the fetus. The diaphragm is forced down and its movements paralyzed by the female holding her breath.

The other respiratory muscles are likewise unable to act, and, hence, imperfect oxidization of the blood results. As a consequence, the cardiac movements are accelerated, greater resistance is met with in the pulmonary and aortic circulations. Moreover, a tendency exists to venous congestion, as evinced by the hue on her face and swollen veins.

Owing to the excruciating pain experienced when the head passes through the cervix, the parturient is further tempted to make additional muscular efforts, which only augment the difficulties met with. Under normal conditions this strain is of such brevity that it cannot be considered of any importance, but when complicated by disease of the heart it is of far greater gravity. If the condition be one of fatty degeneration due to a previous peri- or myocarditis, resulting in faulty nutrition and enfeeblement of the heart's action, as evinced by weak impulse, venous stasis, confused and irregular sounds, anemia alike of the brain and other organs, with faintness and oppression on the slightest exertion, this interference with circulation and respiration may readily tax its powers too far, and so cause speedy death from paralysis. Here the conditions which pertain in surgical anesthesia are absent. The indications present are to allay excessive muscular action and respiratory spasm which is threatening the over-stimulated heart.

To allow the woman to continue such efforts is to permit her to commit suicide; to warn her to desist is useless when in such agony ; while delay is likely to be fatal. How can we overcome this condition of nervous excitement ? Can we accomplish it by the administration of chloroform ? Yes ; of the two evils, for we must acknowledge that there is an element of risk in giving chloroform, we can only choose the lesser, and so promptly proceed by inhalation to relieve the accessory muscles of parturition of their strain. By the abolishment of pain we lessen the work required of the laboring heart, which, instead of beating at the rate of 140 or more a minute, may diminish in frequency to 90 or 100.

What has been said of fatty heart is equally applicable to conditions of hypertrophy and dilatation.

The equilibrium, if disturbed, is almost certain to result disastrously. That sense of fulness in the chest and oppression due to bronchial congestion, if relief is not afforded, becomes most distressing. The cyanosis from deficient aëration is greatly exaggerated, while the insufficient blood-supply to the brain causes syncope, and may be succeeded by coma, if the excessive reflex disturbance be not removed. Nor are the indications for the administration of chloroform materially different in the case of females in labor with valvular disease. Whether it be mitral in the young adult or aortic in the aged primipara, the cardiac strain must be relieved if we would save our patient. As is well known, all forms of valvular disease ultimately develop a condition of ischemia on one side with corresponding low tension, while on the other side is stasis with high tension. While by compensation life may run on for many years, yet, when the strain of parturition comes, it will soon be overthrown if precautions are not taken to prevent it.

Of what benefit will be our knowledge of the value of cardiac "physiological rest," as laid down by Fothergill, if we do not apply it under these conditions? We all appreciate the importance of securing "quietude of mind and body" when such pathological states exist. Then, why not employ the quickest and safest means to obtain it by the inhalation of chloroform? If the danger is great from "active exercise,—climbing mountains, running upstairs, lifting heavy bodies, and all kinds of exercise involving heart strain,"—how much greater, aye, how immeasurably so, must it be when the parturient female forces, with the anguish of despair, every muscle to its utmost in her desire to deliver her child. From a study of chloroform anesthesia in obstetric practice we have seen how it should be administered and how it acts. Surely none will deny that in its employment under these circumstances we act otherwise than for the best interest and safety of our patient. That one may not be charged with being a blind adherent to theory, one has only to turn for support and justification to the teachings of the late lamented Fordyce Barker, who states:

> It seems to be almost accepted as an axiom, with both the profession and public, that the inhalation of chloroform is dangerous for any woman with disease of the heart. For more than thirty years I have been convinced that this opinion is quite erroneous, and I have so taught in my lectures and in former writings.

He goes on further to say: "I have seen several cases, complicated by dangerous heart lesions, which terminated favorably, as I think, solely from the use of chloroform."

Snow, likewise, is of this opinion, "In all forms of valvular disease," he says, "chloroform, when carefully administered, causes less disturbance of the heart and circulation than does severe pain." To quote from Championnière: "If," he says, "I recognized an organic affection of the heart, without pulmonary complications, I should rather give the woman chloroform than to let her suffer." Were further proof necessary as to the propriety of employing chloroform anesthesia, one might include among this group of clinical observers, Vergeley, who expresses himself thus: "Diseases of the heart are not a contra-indication to the use of anesthesia." Macdonald states: "In almost all cases of heart disease with labor, chloroform has been given, and apparently with benefit, during delivery. If carefully administered, I think, it cannot but be useful in all cases." Since such eminent authorities advocate its employment, can we justify ourselves in refusing our patients the benefit and comfort this agent affords? What is the danger from chloroform compared to the state of exhaustion and collapse into which the parturient female will inevitably fall? If this heart is forced to the verge of paralysis from overwork and excitement, why shall we not use the means at our command to lessen that strain? Let us have a reason for the faith that is in us, and not hesitate to fearlessly employ extreme measures to overcome extreme dangers.

Chloroform, by inhalation, can, and will, if properly administered, save the lives of parturient females suffering from organic disease, when death seems imminent from over-stimulation of its ganglia through reflex nervous action. Organic heart disease, then, does not preclude the use of chloroform in labor, but rather is a condition calling for its careful administration.

---

Aristol for Bed-sores.—Dr. J. W. Brooke (*Medical Bulletin*), reports the most gratifying effect obtained by him from the use of aristol for bed-sores in a woman aged sixty-two years, who was confined to bed by a fracture of the femur. The condition had existed for some time, and in spite of every precaution, sloughs began to form on both buttocks. An ointment of forty grains of aristol to an ounce of cosmoline, spread on old linen, was applied, with the result that within forty-eight hours all pain disappeared, and within a week all inflammation was allayed.—*Medical Fortnightly.*

